# Individual feedback on risk for acquiring SARS-CoV-2 infection failed to change future risk behaviors during the COVID-19 pandemic in Japan

**DOI:** 10.1016/j.ssmph.2026.101931

**Published:** 2026-05-11

**Authors:** Shuko Takahashi, Masaru Nohara, Ichiro Kawachi

**Affiliations:** a Teikyo University Graduate School of Public Health, Itabashi, Tokyo, Japan; b Department of Health and Welfare, Iwate Prefectural Government, Morioka, Iwate, Japan; c Department of Social and Behavioral Sciences, Harvard. T.H. Chan School of Public Health, Boston MA, USA

## Abstract

• The effect of receiving personalized risk assessments on changes in preventive behaviors across four analytic samples during the COVID-19 pandemic in Japan.
• Individuals who requested and received feedback on their risk of contracting COVID showed significantly higher ORs of transitioning to high risk in February and April 2021.
• Individuals who received feedback were more likely to improve to lower risk in July and October 2021.
• However, they were less likely to improve to low risk in October 2021 and January 2022.
• Feedback of individualized risk assessments was not associated with improvements in individual behavioral risk behaviors during the pandemic.

## 1 Introduction

The COVID-19 pandemic started in China in 2019 and rapidly spread to the rest of the world. Five years have now passed, and more than 700 million cases of COVID-19 have occurred while the total deaths toll reached 7,084,023 around the world as of January 2025 (World Health Organization, 2025). Countries adopted a range of counter-measures based on available scientific evidence. While some measures contributed to mitigating the spread of COVID-19, other measures were less effective, particularly in the realm of risk communication (Abu-Akel et al., 2021; Kuipers et al., 2021; Richmond et al., 2023).

The adoption of preventive behaviors to stop the spread of infection remains a cornerstone in pandemic control. To promote appropriate actions by each individual, governments took steps such as declaring a state of emergency and providing updated information to guide individual behaviors. In some cases, counter-measures included providing each individualized feedback on risk levels for acquiring infection. For example, a risk assessment tool was introduced in Milwaukee, U.S., to assess COVID-19 mortality risk as a function of personal and neighborhood characteristics (Keval et al., 2023). In Iwate Prefecture, Japan, the local government invited residents to sign up for a risk assessment tool on LINE (a popular messaging app in Japan) from late 2020 to early 2022 (Takahashi et al., 2022, 2024). Each individual's risk of acquiring SARS-CoV-2 infection was calculated using a quantitative assessment tool (the microCOVID)(Takahashi et al., 2022) which classified each individual's risk level into low, middle, and high risk for acquiring infection. The government then provided individualized feedback to respondents to guide their behavior.

According to classical theories of behavioral intervention, such as the Health Belief Model, individuals may lack motivation to change their behavior because of inaccurate beliefs about their individual susceptibility to disease. Accordingly, the goal of health communication should be to provide accurate information about disease susceptibility and severity (Cipolletta et al., 2022). Similarly, Protection Motivation Theory explains how people adopt protective behaviors based on threat appraisal and their ability to cope with the threat (Rogers, 1975). The theory has been applied in several studies as a framework for examining the relationship between perceived risk and preventive behaviors during the COVID-19 pandemic. (Ezati Rad et al., 2021; Hinssen & Dohle, 2023). However, research specifically focusing on providing individualized feedback on personal risk assessments remains limited.

We hypothesized that individuals who received feedback on their risk level would exhibit positive shifts in their future preventive behavior. Specifically, those who were informed that their prior survey responses indicated high-risk for acquiring SARS-Cov-2 infection would experience an increase in perceived risk via cognitive appraisal processes, including perceived vulnerability and perceived severity. In turn, we hypothesized that heightened threat perception would lead to lower-risk behaviors in the next survey wave compared to those who did not request or receive feedback. Accordingly, the aim of this study is to prospectively examine whether providing feedback on behavioral risk assessments influenced individuals' behaviors across five sequential surveys conducted during the COVID-19 pandemic in Iwate Prefecture, Japan.

## 2 Methods

### 2.1 Study population

Iwate Prefecture is located in northern Japan and has an area of 15,280 km^2^, with a population of some 1.2 million. The total number of COVID-19 cases as of May 9, 2023, was 238,133, including 625 COVID-19-related deaths (NHK, 2024).

### 2.2 Data

The Iwate Prefectural Government conducted serial cross-sectional surveys of residents in Iwate Prefecture using a social network platform “LINE” (LINE Corporation, Tokyo, Japan) since the early phase of the pandemic (started in December 2020). Iwate Prefecture reported information to users relating to the pandemic, including the number of newly confirmed cases and the number of cumulative deaths. The platform was also used to conduct a series of surveys every two months to capture the residents’ preventive behavior and perception of risk for SARS-CoV-2. Messages were posted through the platform inviting registered residents to participate in the surveys.

An online questionnaire was presented on the site where approximately 170,000 people had been registered at the time of the survey. We used the data collected from the second to sixth survey waves of registered people (the second survey wave, from February 5 to 7, 2021; the third survey wave, from April 16 to 18, 2021; the fourth survey wave, from July 2 to 4, 2021; the fifth survey wave, from October 1 to 3, 2021; the sixth survey wave, from January 7 to 10, 2022). Responses were received from 18,724, 30,165, 24,230, 22,776, and 27,790 individuals in the second survey wave, in the third survey wave, in the fourth survey wave, in the fifth survey wave, and in the sixth survey wave (response rate ∼ 16.3%). After excluding subjects who were lost to follow-up by the next wave, those who lived outside Iwate, and those who were working in the healthcare services (and therefore already had a high level of awareness of contracting infection), we received responses from 9385 individuals who answered in both the second and third survey waves (men, 33.2%), 11,907 individuals (men, 32.8%) who answered in both the third and fourth survey waves, 9635 individuals (men, 31.5%) who answered in both the fourth and fifth survey waves, and 10,636 individuals (men, 34.7%) who answered in both the fifth and sixth survey waves (Supplementary Fig. 1).

### 2.3 Outcome

Each individual's risk of contracting SARS-CoV-2 infection was calculated using a quantitative assessment tool called “the microCOVID” (microCOVID Project, 2020). MicroCOVID is a calculator to numerically quantify people's risk of acquiring COVID-19 by asking them about their behavior over the past week. MicroCOVIDs were assessed by using three main domains: activity risk, person risk, and number of people with whom an individual interacts. We calculated the microCOVID's value for each person using the score = activity risk x number of people x person risk. The unit of 1.0 microCOVID indicates a one-in-a-million chance of contracting COVID. The details of the microCOVID risk assessment have been published in the previous paper (Takahashi et al., 2022). The score of the microCOVID classified each individual's risk level into low, middle, and high risk for contracting infection. We grouped them into either “low risk” (low-risk group, <25 microCOVIDs) or “high risk” of infection (middle and high-risk group, ≥ 25 microCOVIDs). After grouping individual risk levels into two groups, we calculated trajectories of behavior change across survey waves.

### 2.4 Covariates

The questionnaires in each survey were asked about the individual's age, sex, municipality of residence, and occupation. Respondents selected one age group from seven groups: “under 20 years,” “aged 20–29 years,” “aged 30–39 years,” “aged 40–49 years,” “aged 50–59 years,” “aged 60–69 years,” and “age 70 years old or older.” We grouped them into three age groups: young (people under 40 years of age), middle-aged (people aged 40 to 59 years), and elderly (people aged 60 years or older). Residential areas were classified into two categories, inland and coastal/mountainous, based on the geographic and population activity characteristics of the Prefecture. Occupation was assessed with the question, “What is your current job?” Participants selected one of the 11 occupations: transportation, customer-facing occupations in the retail/hospitality sector, office workers, managers in customer-facing industries, advertising or media, workers in manufacturing, unemployed including homemakers, healthcare workers, government workers, teachers, students, farmers/agricultural workers, and workers in other jobs. After excluding healthcare workers, respondents were grouped into the following four occupational groups: workers in service industries (transportation, customer-facing occupations in the retail/hospitality sector, or office workers), education sector (teachers or students), government workers, and all others (workers in manufacturing, farmers/agricultural workers, workers in other jobs, or unemployed).

After answering items related to microCOVID risk assessment, each respondent was asked “Would you like to receive feedback on the results of your risk assessments calculated from your survey responses?” If the respondents chose “Yes”, we provided the results of their behavioral risks approximately one week later after the survey while we did not provide the results of individual assessments if they chose “No”. Based on the results, participants were divided into two groups: received feedback and did not receive feedback.

### 2.5 Statistical analyses

To identify the trajectories of behavioral changes across survey waves, we defined four analytical groups of respondents. For example, the first group included subjects who participated in both the second and third survey waves, the second group were those who participated in both the third and fourth survey waves, and so on. To identify the trajectories of behavioral risks in each group, respondents were classified into four categories: (1) persistent low risk—individuals classified as low risk in the prior survey & who remained low risk in the next survey; (2) improved to low risk—individuals classified as high risk in the prior survey who later transitioned to low risk; (3) increased to high risk—individuals classified as low risk in the prior survey who later transitioned to high risk; and (4) persistent high risk—individuals classified as high risk in the prior survey who remained high risk in the next survey. Baseline characteristics were compared for the four trajectories in each group using the chi-squared test or Fisher's exact test. Multinomial logistic regression was used to assess whether individual feedback was associated with behavioral change. Two logistic regression models were constructed to control for potential confounding variables in the relationship between receiving feedback and behavioral changes. Model 1 was adjusted for age and sex, while Model 2 additionally accounted for occupation and residential area.

We also conducted multiple imputation as sensitivity analyses, generating five imputed datasets by imputing missing covariate data using the Markov Chain Monte Carlo method. All analyses were performed using the Statistical Package for the Social Sciences (SPSS) software, version 25.0 (IBM, Chicago, IL, USA). All statistical tests were two-sided, and P-values <0.05 were considered statistically significant.

## 3 Results

Table 1a, Table 1b, Table 1c, Table 1d shows the baseline characteristics of participants in the persistent low-risk group, improved to low-risk group, increased to high-risk group and persistent high-risk group for the four analytical samples. The number of individuals who exhibited persistent low risk was small across all analytical samples. Overall, younger individuals were more likely to be classified in the persistent high-risk group across all analytical samples. Notably, they were also overrepresented in the group that increased to high risk in the first and fourth analytic samples. While the proportion of middle-aged individuals was highest in the “improved to low-risk” group in the second analytic sample (64.7%), the prevalence of older adults was highest in the increased to high-risk group (26.9%). Additionally, women exhibited higher engagement in risky behaviors compared to men throughout the study period. Regarding occupation, workers in the service industry were more likely to be classified in the “increased to high-risk” group in the third analytic sample (43.3%) but were less likely to be in the improved to low-risk group in the fourth analytic sample (34.1%). By contrast, individuals in the education sector (teachers and students) showed the opposite pattern, with a low proportion in the “increased to high-risk” group in the third analytic sample (3.8%) but a high proportion in the “improved to low-risk” group in the fourth analytic sample (34.1%). Additionally, the prevalence of receiving feedback declined in the improved to low-risk group in the fourth analytic sample (68.3%), with a significant difference observed only in the final analytic sample (from the fifth to sixth survey waves).

**Table 1a:** Baseline characteristics of participants in the first group between in the second and third surveys waves (n = 9385).

		Missing	Persistent low risk (n = 8612)	Improved to low risk (n = 283)	Increased to high risk (n = 354)	Persistent high risk (n = 136)	
		n (%)	n (%)	n (%)	n (%)	n (%)	*P* value
**Age groups**	**Young**	0 (0.0)	2168 (25.2)	100 (35.3)	122 (34.5)	47 (34.6)	<0.001
	**Middle age**		4924 (57.2)	156 (55.1)	210 (59.3)	84 (61.8)	
	**Elderly**		1520 (17.6)	27 (9.5)	22 (6.2)	5 (3.7)	
**Sex**	**Men**	39 (0.4)	2862 (33.4)	98 (34.6)	116 (33.0)	41 (30.1)	0.834
	**Women**		5713 (66.6)	185 (65.4)	236 (67.0)	95 (69.9)	
**Occupation**	**Government workers**	0 (0.0)	1158 (13.4)	43 (15.2)	60 (16.9)	28 (20.6)	<0.001
	**Service industries**		3031 (35.2)	128 (45.2)	147 (41.5)	58 (42.6)	
	**Education sector**		831 (9.6)	54 (19.1)	86 (24.3)	36 (26.5)	
	**All other**		3592 (41.7)	58 (20.5)	61 (17.2)	14 (10.3)	
**Residential areas**	**Inland areas**	0 (0.0)	6970 (80.9)	220 (77.7)	271 (76.6)	115 (84.6)	0.069
	**Coastal and mountainous areas**		1642 (19.1)	63 (22.3)	83 (23.4)	21 (15.4)	
**Receive feedback**	**Receiving feedback**	0 (0.0)	7428 (86.3)	239 (84.5)	315 (89.0)	109 (80.1)	0.065
	**Not receiving feedback**		1184 (13.7)	44 (15.5)	39 (11.0)	27 (19.9)	

Categorical variables are presented as number of cases (%).

*P* values were calculated using the chi-squared test.

**Table 1b:** Baseline characteristics of participants in the second group between in the third and fourth surveys waves (n = 11,907).

		Missing	Persistent low risk (n = 10,405)	Improved to low risk (n = 493)	Increased to high risk (n = 956)	Persistent high risk (n = 53)	
		n (%)	n (%)	n (%)	n (%)	n (%)	*P* value
**Age groups**	**Young**	0 (0.0)	1930 (18.5)	137 (27.8)	177 (18.5)	18 (34.0)	<0.001
	**Middle age**		5904 (56.7)	319 (64.7)	522 (54.6)	31 (58.5)	
	**Elderly**		2571 (24.7)	37 (7.5)	257 (26.9)	4 (7.5)	
**Sex**	**Men**	71 (0.6)	3498 (33.8)	163 (33.4)	326 (34.4)	12 (22.6)	0.374
	**Women**		6848 (66.2)	325 (66.6)	623 (65.6)	41 (77.4)	
**Occupation**	**Government workers**	0 (0.0)	1157 (11.1)	76 (15.4)	104 (10.9)	5 (9.4)	<0.001
	**Service industries**		3685 (35.4)	215 (43.6)	336 (35.1)	25 (47.2)	
	**Education sector**		865 (8.3)	128 (26.0)	85 (8.9)	13 (24.5)	
	**All other**		4698 (45.2)	74 (15.0)	431 (45.1)	10 (18.9)	
**Residential areas**	**Inland areas**	0 (0.0)	8308 (79.8)	401 (81.3)	782 (81.8)	35 (66.0)	0.028
	**Coastal and mountainous areas**		2097 (20.2)	92 (18.7)	174 (18.2)	18 (34.0)	
**Receive feedback**	**Receiving feedback**	0 (0.0)	8984 (86.3)	435 (88.2)	818 (85.6)	47 (88.7)	0.526
	**Not receiving feedback**		1421 (13.7)	58 (11.8)	138 (14.4)	6 (11.3)	

Categorical variables are presented as number of cases (%).

*P* values were calculated using the chi-squared test or Fisher's exact test.

**Table 1c:** Baseline characteristics of participants in the third group between in the fourth and fifth surveys waves (n = 9635).

		Missing	Persistent low risk (n = 8908)	Improved to low risk (n = 614)	Increased to high risk (n = 104)	Persistent high risk (n = 9)	
		n (%)	n (%)	n (%)	n (%)	n (%)	*P* value
**Age groups**	**Young**	0 (0.0)	1849 (20.8)	190 (30.9)	15 (14.4)	5 (55.6)	<0.001
	**Middle age and elderly**		7059 (79.2)	424 (69.1)	89 (85.6)	4 (44.4)	
**Sex**	**Men**	48 (0.5)	3107 (35.1)	202 (33.1)	36 (34.6)	3 (33.3)	0.812
	**Women**		5757 (64.9)	408 (66.9)	68 (65.4)	6 (66.7)	
**Occupation**	**Government workers**	0 (0.0)	985 (11.1)	103 (16.8)	7 (6.7)	2 (22.2)	<0.001
	**Service industries**		3228 (36.2)	272 (44.3)	45 (43.3)	4 (44.4)	
	**Education sector**		760 (8.5)	126 (20.5)	4 (3.8)	2 (22.2)	
	**All other**		3935 (44.2)	113 (18.4)	48 (46.2)	1 (11.1)	
**Residential areas**	**Inland areas**	0 (0.0)	7124 (80.0)	502 (81.8)	85 (81.7)	6 (66.7)	0.505
	**Coastal and mountainous areas**		1784 (20.0)	112 (18.2)	19 (18.3)	3 (33.3)	
**Receive feedback**	**Receiving feedback**	0 (0.0)	7232 (81.2)	512 (83.4)	88 (84.6)	6 (66.7)	0.278
	**Not receiving feedback**		1676 (18.8)	102 (16.6)	16 (15.4)	3 (33.3)	

Categorical variables are presented as number of cases (%).

*P* values were calculated using the chi-squared test or Fisher's exact test.

**Table 1d:** Baseline characteristics of participants in the fourth group between in the fifth and sixth surveys waves (n = 10,636).

		Missing	Persistent low risk (n = 9352)	Improved to low risk (n = 41)	Increased to high risk (n = 1192)	Persistent high risk (n = 51)	
		n (%)	n (%)	n (%)	n (%)	n (%)	*P* value
**Age groups**	**Young**	0 (0.0)	1590 (17.0)	14 (34.1)	339 (28.4)	19 (37.3)	<0.001
	**Middle age**		5279 (56.4)	23 (56.1)	706 (59.2)	27 (52.9)	
	**Elderly**		2483 (26.6)	4 (9.8)	147 (12.3)	5 (9.8)	
**Sex**	**Men**	55 (0.5)	3217 (34.6)	16 (39.0)	365 (30.8)	24 (47.1)	0.013
	**Women**		6088 (65.4)	25 (61.0)	819 (69.2)	27 (52.9)	
**Occupation**	**Government workers**	0 (0.0)	1064 (11.4)	7 (17.1)	197 (16.5)	5 (9.8)	<0.001
	**Service industries**		3094 (33.1)	14 (34.1)	474 (39.8)	21 (41.2)	
	**Education sector**		904 (9.7)	14 (34.1)	168 (14.1)	8 (15.7)	
	**All other**		4290 (45.9)	6 (14.6)	353 (29.6)	17 (33.3)	
**Residential areas**	**Inland areas**	0 (0.0)	7379 (78.9)	31 (75.6)	962 (80.7)	42 (82.4)	0.439
	**Coastal and mountainous areas**		1973 (21.1)	10 (24.4)	230 (19.3)	9 (17.6)	
**Receive feedback**	**Receiving feedback**	0 (0.0)	7838 (83.8)	28 (68.3)	990 (83.1)	45 (88.2)	0.039
	**Not receiving feedback**		1514 (16.2)	13 (31.7)	202 (16.9)	6 (11.8)	

Categorical variables are presented as number of cases (%).

*P* values were calculated using the chi-squared test or Fisher's exact test.

**Fig. 1:**
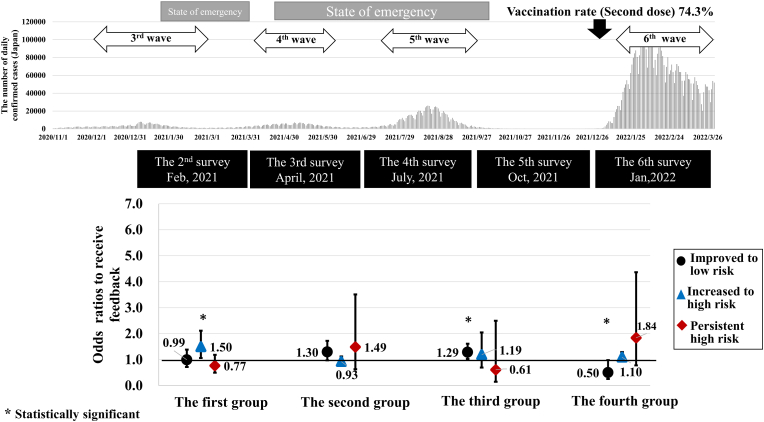
Results of the analysis using models for risk trajectories across four analytic samples in sequential survey waves.

Fig. 1 shows the effect of receiving individualized feedback on changes in risk behaviors during the COVID-19 pandemic. Individuals who received feedback on their risk of contracting COVID-19 had significantly *higher* odds ratios (*ORs*) of transitioning to high risk in the first analytic sample, i.e., between the second and third survey waves conducted in February and April 2021 (*OR* 1.50, 95% confidence interval (95% *CI*): 1.06, 2.12) (Supplementary Table 2). Although those who received feedback were more likely to transition to low risk in the third analytic sample between the fourth and fifth survey waves conducted from July to October 2021, (*OR* 1.29, 95% *CI*: 1.03, 1.62), they were subsequently less likely to transition to low risk in the fourth analytic sample (between the fifth and sixth survey waves conducted from October 2021 to January 2022) (*OR* 0.50, 95% *CI*: 0.26, 0.98).

In the sensitivity analyses using multiple imputation for missing covariates, similar results were observed as in the main analyses (Supplementary Table 3). Additionally, the *ORs* for “improved to low risk” was significant in the second analytic sample, obtained between the third and fourth survey waves conducted in April and July 2021. (*OR* 1.31, 95% *CI*: 1.13, 1.52).

## 4 Discussion

We examined the effect of receiving personalized risk assessments on changes in preventive behaviors across four analytic samples. Contrary to our hypothesis, the intervention did not produce consistent results. Specifically, receiving feedback had a substantial effect in increasing the adoption of preventive behavior during the middle period of the pandemic, from July to October 2021 (the third group between the fourth and fifth survey waves), while it negatively impacted preventive behavior in the first group between the second and third survey waves, from February to April 2021, and in the fourth group, from October 2021 to January 2022. Nevertheless, these analyses provide meaningful insights into the impact of individualized risk feedback and its potential effectiveness in influencing future behaviors during the COVID-19 pandemic.

A few studies have been reported about relationship between providing individualized feedback and behavioral changes. Sinclair et al. examined changes in subjective perceived risk regarding COVID-19 and engagement in risky behaviors by providing feedback on the accuracy of participants' risk estimation in an episodic simulation task conducted in 2020 (*n* = 735, USA) (Sinclair et al., 2021). They identified lasting increases in perceived risk and decreases in willingness to engage in risky activities after a delay, across different feedback conditions. The focus of their study on the effectiveness of feedback was similar to ours, but their analysis was limited to the early stages of the pandemic. By contrast, our study assessed the effectiveness of providing feedback across multiple time-points as the pandemic progressed. Furthermore, the content of the feedback differed in the study by Sinclair et al. (2021) compared to ours; they estimated the probability of certain events in participants' locations and provided veridical feedback about risk probabilities. In our study, however, we numerically calculated each individual's current risk based on various factors, including mask-wearing, distance from others, and other behaviors, and provided feedback based on these calculated risks.

Sinclair et al. also conducted a similar survey during the Omicron variant outbreak (*n =* 11,169, USA) (Sinclair et al., 2023). This study was similar to ours in terms of feedback interventions. However, the methods of risk assessment and the survey periods differed. While participants in their study received feedback on the accuracy of their infectious risk scores estimated in hypothetical situations, with a focus on the change in willingness to participate in various events over a four-month period, our study provided feedback based on quantitative risk assessments and evaluated behavioral changes across four different periods during five sequential surveys conducted over 17 months. The repeated assessments of individuals' behavioral risks with feedback during the COVID-19 pandemic allowed us to evaluate the effectiveness of receiving information about their behavioral risks over time.

Protection Motivation Theory emphasizes two components that affect behavioral adoption: threat appraisal and coping appraisal. These components are influenced by environmental sources of information, such as culture and ideology, interpersonal sources of information, such as personal experience, and individual demographic factors. The emergence of effective vaccines undoubtedly led to some impacts on threat appraisal and coping appraisal. Although the external environment was changing, we initially hypothesized that receiving individualized risk assessments—allowing individuals to understand their actual risks—would lead to increased adherence to appropriate behaviors through threat appraisal and emotional responses such as fear or anxiety. However, consistent results were not observed. While it is unsurprising that awareness of infection declines as the pandemic progresses, our findings revealed contrapositive results even in the early phase of the pandemic, particularly in the first group between the second and third survey waves conducted from February to April 2021.

The results indicate that a high risk perception does not necessarily lead to behavior change, as suggested by the Protection Motivation Theory framework. Several underlying factors may explain this discrepancy. First, regarding our study design, the timing of our initial survey may provide some insights. Since we did not conduct a baseline survey prior to the pandemic, we were unable to assess changes in behavior from before to the middle of the pandemic. Additionally, the concept of 'pandemic fatigue'—which describes the decline in motivation to engage in protective behaviors over time—has been reported in several countries and may have influenced our findings (Kim et al., 2023; Petherick et al., 2021). The results of individuals in the low-risk group during the fifth survey who later transitioned to high risk in the sixth survey in January 2022 suggest this possibility. By that time, participants had been engaged in high-level preventive behaviors for several years since the onset of the pandemic and may have experienced a behavioral rebound due to prolonged restrictions. Although we did not include items specifically designed to measure ’pandemic fatigue’, the pattern of increased risk-taking behaviors in later waves suggested that prolonged public health crises can lead to decreased compliance due to fatigue. Second, psychological distancing among racial or cultural groups could also be a contributing factor. For example, Skinner-Dorkenoo et al. highlighted that the disproportionate impact of COVID-19 on Black communities led some White U.S. residents to perceive the virus as less threatening to themselves (Skinner-Dorkenoo et al., 2022). When individuals compare themselves to others who are less well off, they may feel more satisfied with their own situation. In a politically polarized country such as the United States, communicating facts about the severity of diseases for one ethnic group can sometimes be counterproductive. However, since Japan does not have racial disparities to the same extent, this explanation may not be directly applicable. Nonetheless, a similar phenomenon may have occurred due to hierarchical comparisons, such as occupational disparities. In the early phase of the pandemic, breaking news frequently highlighted COVID-19 outbreaks among service industry workers, even as the government urged the public to refrain from drinking and social gatherings (Seiyo City in Ehime Japan, 2020). As other occupational groups perceived themselves as different from service industry workers, they may have received a sense of (false) reassurance about their own circumstances, which could have contributed to riskier behavior. Third, the timing and frequency of feedback may play a role in health behavior change. Regarding the impact of feedback timing on behavior modification, a systematic review suggests that continuously available, personalized, and actionable feedback is more effective in promoting behavior change, particularly in areas such as diet and physical activity (Schembre et al., 2018). A one-week timeframe for providing feedback on behavioral assessments may have had a weaker effect on modifying behavior. Finally, an individual's decision to receive feedback may influence subsequent behavior change. Studies suggest that choice-based interventions are associated with higher adherence compared to interventions that do not offer a choice (Carlisle et al., 2022). We anticipated that individuals who chose to receive their risk assessments would exhibit increased preventive behaviors; however, the results did not reveal consistent patterns. While self-determination is crucial in motivating human behavior, its effectiveness may have been diminished by various external factors in the unique and unpredictable context of the COVID-19 pandemic.

We found a significant increase in the ORs for transitioning to high risk in the first analytic sample (February to April 2021). Several factors may explain this finding. Following the third COVID-19 wave (December 2020–January 2021), a second state of emergency was in place from January 8 to March 21, 2021, mainly in densely populated areas such as Tokyo. These measures included requests for restaurants to shorten business hours and for residents to refrain from going out. The second survey wave (February 2021) was conducted during this period of strict restrictions. However, as infection numbers gradually decreased, adherence to preventive measures may have weakened by the time of the third survey (April 2021). In addition, the national vaccination program began in February 2021, which may have slightly lowered perceived risk. Although these external factors were not included in our statistical models, they may explain the observed increase in high-risk behaviors despite the provision of individualized behavioral risk feedback.

Our study demonstrated that recognizing the risk of infection does not consistently lead to appropriate preventive actions. Simply providing numerical information, such as the number of COVID-19 cases or the declaration of a state of emergency, may have limited effectiveness, making policy interventions challenging to implement. Individual risk perception is influenced by various factors, including emotions and personal experiences, complicating risk communication efforts aimed at matching perceived risk levels to actual risk situations (Brown, 2014; Savadori et al., 2022). In turn, individuals do not passively receive risk information nor respond purely rationally, as their reactions are influenced by social context and their level of trust in the information source (Alaszewski, 2005). Furthermore, the COVID-19 pandemic spanned three years globally, during which people's risk perception inevitably changed over time (Abir et al., 2020), the complex interplay between public health messaging, risk perception, and behavior throughout the pandemic highlights the need for inclusive communication strategies. Policymakers should emphasize the universal nature of infectious risks to encourage widespread adherence to preventive measures.

## 5 Limitations

Our study highlights the complexity of the relationship between risk perception and preventive behavior. However, there are several important considerations when interpreting our results. First, some key variables were not assessed in this survey, including current employment status, job position, household income, years of education, and individual health status since we could not avoid limiting the number of questions, which leaded exclude certain key variables in order to obtain valid responses and a sufficient number of respondents during the pandemic. Second, we could not include external factors—such as government interventions or media coverage—that may have influenced participants' behavioral changes, as these were difficult to quantify. Including such variables could have allowed for a more precise estimation of the effect of receiving feedback on behavioral changes. Third, selection bias may be a concern, as individuals lost to follow-up were observed (the percentage of survey loss was 37.2% between the second and third survey waves, 50.3% between the third and fourth waves, 49.5% between the fourth and fifth waves, and 42.0% between the fifth and sixth waves). We compared the demographic characteristics of those who participated in the prior survey but did not participate in the subsequent survey with those who participated in both survey waves **(**Supplementary Table 4). As non-participants were more likely to be young men, workers in the service industry, and individuals who did not receive behavioral risk assessments, our analysis may have been biased toward middle-aged and elderly women, who were more likely to receive their behavioral risk assessments. To consider possible selection bias caused by loss to follow-up, we calculated the *ORs* between the people who participated in the prior survey but did not participate in the subsequent survey with those who participated in both survey waves using inverse propensity weighting in the same analyses (Supplementary Table 5). Similar results were observed as in the main findings. Fourth, caution is needed when generalizing the results since the response rate in the present survey was low. A comparison of demographic characteristics between our participants and census data during the final analytic period showed that the survey participants were more likely to be middle-aged women compared to the census population (Supplementary Table 6).Fifth, the microCOVID tool itself has not been formally validated in Japan. As being careful to input Japan-specific behavioral and epidemiological data, such as mask adherence rates, mobility patterns, and household contact structure, this contextual adaptation allows for culturally relevant modeling while maintaining consistency with the tool's original framework. Finally, the feedback we conducted in this survey did not assess comprehension or perceived usefulness of the messages.

## 6 Conclusion

On the whole, individualized feedback on risk was not associated with improvements in individual behavior during the pandemic and, in some cases, appeared to have the opposite effect. A key lesson from this study is that simply providing people with accurate risk assessments does not necessarily lead to behavioral change. Policymakers should consider the complexity of risk communication when promoting effective measures and developing strategies for risk communication.

## CRediT authorship contribution statement

**Shuko Takahashi:** Writing – original draft, Funding acquisition, Formal analysis, Conceptualization. **Masaru Nohara:** Writing – review & editing, Supervision, Resources, Project administration. **Ichiro Kawachi:** Writing – review & editing, Validation, Supervision, Methodology.

## Ethics considerations

The research plan was deliberated and approved by the Ethics Committee of Iwate Medical University Institute Review Board (approval no. MH2024-021).

## Declaration of competing interest

S Takahashi involved in SHIONOGI RESEARCH PROMOTION FOUNDATION Grant with SHIONOGI RESEARCH PROMOTION FOUNDATION.

## Data Availability

Data will be made available on request.

## Appendix A Supplementary data

Multimedia component 2

Supplementary Fig. 1. Flow chart of the selection of respondents. Out of 170000 registered residents, Out of 170,000 registered residents, 9,385, 11,907, 9, 635, and 10,636 individuals were selected in the four analytic samples, respectively.Multimedia component 3
