# Supplementary material for: Individual feedback on risk for acquiring SARS-CoV-2 infection failed to change future risk behaviors during the COVID-19 pandemic in Japan
Source: SSM Popul Health. 2026 May 11;34:101931. doi: 10.1016/j.ssmph.2026.101931 (PMC13199801; doi:10.1016/j.ssmph.2026.101931)
Supplement: Multimedia component 1 [file mmc1.docx]

# **Supplementary data**

**Supplementary Text. Detailed calculation of microCOVID.**

The microCOVID project is a calculator to measure and numerically quantify the risk of getting COVID-19 from an activity or relationship in participants' daily life based on available evidence. A score of 1 microCOVID means a one-in-a-million chance of getting COVID. microCOVIDs are computed by using three major factors: activity risk, person risk, and number of people. We obtained the value by multiplying person interactions: microCOVID = activity risk x number of people x person risk for one person.

Activity risk indicates the chance that the activity will transmit COVID-19 from one to the other person who currently has COVID-19. In accordance with a previous study conducted by The Behavioral Insights Team in the UK (Yihan et al., 2020), we modified some items in the questionnaire by asking respondents “Did you meet anyone outside your household (or support bubble) in a risky situation in the past week?”. A microCOVID value of zero was given to respondents who did not meet any person. Respondents who answered that they met someone went to the next questions asked about the details of the microCOVIDs value. Activity risk is a weighting based on coefficients how the interaction in the question is different from the reference value (**Supplementary Table 1**). Coefficients of risk are assigned to other types of interaction which differ from that reference value (if one person who has COVID-19, for 1 hour, indoors, unmasked, at 3 feet, there is a baseline 9% chance of transmission per hour) based on the following factors: duration of interaction, mask wearing (respondents and other persons), indoor/outdoor environment (indoor without ventilation, outdoor, indoor with an air conditioning systems, a train with air filtration, an airplane, a moving car with the windows rolled down, a space with one or more sides open to the outdoors), distance from each other, volume of conversation, and frequency of risky situations (times a week). We calculated activity risk by multiplying 9% in some survey: activity risk = coefficients x 9%.

With regard to the number of people, we asked respondents “How many people were there within a 5-meter radius of the scene?”

Person risk represents the chance that the other person currently has COVID based on overall prevalence in the person’s area and recent behaviors of the person. Because the chance of a person having COVID depends not only on whether that person has any symptoms but also on the actions and choices of that person in the past 10 days, we used three different methods for estimating the probability of a person having COVID: a basic method, an intermediate method and an advanced method. The basic method is just an assumption that a person is “average” for their region. The probability of your friend having COVID is the probability of anyone in their geographic area having COVID. New infections in the past week were calculated as follows: new infections in the past week = reported cases x underreporting factor x delay factor. Based on that value, we estimated the person risk as follows: person risk (basic) = new infections in the past week / population in millions. The intermediate method is a quick adjustment for the risk a person being exposed at his/her workplace. The advance method involves adding up the risk of each individual activity that the person has done recently with consideration of three categories: socializing, errands, and work. In the intermediate and advanced methods, we calculated person risk by multiplying person risk (basic) by weighting coefficients in the following 14 items which are different from each survey) (**Supplementary Table 1**): “being an essential worker (working in health care, transportation or retailing)”, “living alone and only going to grocery stores for shopping”, “living in a closed pod of 4 people”, “living in a closed pod of 10 people”, “living in a closed pod of 20 people”, “having gone to a bar in the past 10 days”, “you had prolonged contact with one person with whom you don't normally get along”, “you had prolonged contact with about four people with whom you don't normally get along”, “you had prolonged contact with about 10 people with whom you don't normally get along”, “you live with essential workers”, “having been a patient with COVID-19”, “you and other persons living with you only going to grocery stores for shopping”, “not have any interaction with others and other persons living with you who are essential workers”, and “not applicable for those items”. In the sixth survey wave, we applied weighting coefficients to vaccination status and multiplied vaccination status with the microCOVID score.

# **References**

Yihan, X., Mark, E., Tania, L., Lev, T., Abigail, M., & Hugo, H. (2020). A small number of people account for a large amount of coronavirus risk. United Kingdom: The behavioural insights team.
