# Supplementary material for: Individual feedback on risk for acquiring SARS-CoV-2 infection failed to change future risk behaviors during the COVID-19 pandemic in Japan
Source: SSM Popul Health. 2026 May 11;34:101931. doi: 10.1016/j.ssmph.2026.101931 (PMC13199801; doi:10.1016/j.ssmph.2026.101931)
Supplement: Multimedia component 2 [file mmc2.docx]

# **Supplementary Data**

**Supplementary Table 1. Weighting coefficients for items within each factor (activity, person risk, and vaccination status) from the second and sixth surveys**

| **Risk** | **Factors** | **Items** | **Coefficient** | | | | |
| --- | --- | --- | --- | --- | --- | --- | --- |
|  |  |  | **The 2^nd^ survey** | **The 3^rd^ survey** | **The 4^th^ survey** | **The 5^th^ survey** | **The 6^th^ survey** |
| **Activity Risk** | **Respondents' mask** | No mask or inappropriate usage | 1 | 1 | 1 | 1 | 1 |
|  |  | Polyester or polyurethane mask | 1 | 1 | 1 | 1 | 1 |
|  |  | Cloth or bandanna | 1 | 1 | 1 | 1 | 1 |
|  |  | Surgical mask, non-woven mask | 0.5 | 0.5 | 0.5 | 0.5 | 0.5 |
|  |  | N95 mask | NA | 0.1 | 0.1 | 0.33 | 0.33 |
|  | **Other person's mask** | No mask or inappropriate usage | 1 | 1 | 1 | 1 | 1 |
|  |  | Polyester or polyurethane mask | 0.5 | 0.5 | 0.5 | 0.5 | 0.5 |
|  |  | Cloth or bandanna | 0.25 | 0.25 | 0.25 | 0.25 | 0.5 |
|  |  | Surgical mask, non-woven mask | 0.25 | 0.25 | 0.25 | 0.25 | 0.25 |
|  |  | N95 mask | 0.1 | 0.1 | 0.1 | 0.17 | 0.17 |
|  | **Indoor/outdoor environment** | Indoor without ventilation | 1 | 1 | 1 | 1 | 1 |
|  |  | Outdoor | 0.05 | 0.05 | 0.05 | 0.05 | 0.05 |
|  |  | Indoor with air conditioning systems | 0.25 | 0.25 | 0.25 | 0.25 | 0.25 |
|  |  | A train with air filtration | 0.25 | 0.25 | 0.25 | 0.25 | 0.25 |
|  |  | An airplane | 0.17 | 0.17 | 0.17 | 0.17 | 0.17 |
|  |  | A moving car with the windows rolled down | 0.25 | 0.25 | 0.25 | 0.25 | 0.25 |
|  |  | A space with one or more sides open to the outdoors | 0.25 | 0.25 | 0.25 | 0.25 | 0.25 |
|  | **Distance from each other** | < 0.3 meters apart | 2 | 2 | 2 | 2 | 2 |
|  |  | About 1 meter apart | 1 | 1 | 1 | 1 | 1 |
|  |  | 2+ meters apart | 0.5 | 0.5 | 0.5 | 0.5 | 0.5 |
|  |  | 3+ meters apart | 0.25 | 0.25 | 0.25 | 0.25 | 0.25 |
|  | **Volume of conversation** | Not talking | 0.2 | 0.2 | 0.2 | 0.2 | 0.2 |
|  |  | Normal conversation | 1 | 1 | 1 | 1 | 1 |
|  |  | Loud talking (shouting, talking over music, singing) | 5 | 5 | 5 | 5 | 5 |
| **Person Risk** | **Intermediate and Advanced Method** | Being an essential worker | 2 | 2 | 2 | 1 | 1 |
|  |  | Living alone and only going to grocery stores for shopping | 0.011 | 0.011 | 0.011 | 0.023 | 0.023 |
|  |  | Living in a closed pod of 4 people | 0.021 | 0.021 | 0.021 | 0.05 | 0.05 |
|  |  | Living in a closed pod of 10 people | 0.041 | 0.041 | 0.041 | 0.11 | 0.11 |
|  |  | Living in a closed pod of 20 people | 0.075 | 0.075 | 0.075 | 0.2 | 0.2 |
|  |  | Having gone to a bar in the past 10 days | 14.5 | 14.5 | 14.5 | 22.73 | 22.73 |
|  |  | You had prolonged contact with one person with whom you don't normally get along. | NA | NA | NA | 0.43 | 0.43 |
|  |  | You had prolonged contact with about four people with whom you don't normally get along. | NA | NA | NA | 1.23 | 1.23 |
|  |  | You had prolonged contact with about 10 people with whom you don't normally get along. | NA | NA | NA | 3.64 | 3.64 |
|  |  | You live with essential workers. | NA | 0.61 | 0.61 | 0.82 | 0.82 |
|  |  | Having been a patient with COVID-19 | 1 | 1 | 1 | NA | NA |
|  |  | You and other persons living with you only going to grocery stores for shopping | 0.014 | 0.014 | 0.014 | 0.032 | 0.032 |
|  |  | Not have any interaction with others and other persons living with you who are essential workers | 0.61 | 0.61 | 0.61 | NA | NA |
|  |  | Not applicable for those items | 1 | 1 | 1 | 1 | 1 |
| **Vaccination status** | **The number of vaccinations** | Zero dose of vaccination | NA | NA | NA | NA | 1 |
|  |  | One dose of vaccination | NA | NA | NA | NA | 0.76 |
|  |  | Two or more dose of vaccination | NA | NA | NA | NA | 0.17 |
|  |  | Others | NA | NA | NA | NA | 1 |

**Supplementary Table 2. Results of analysis for risk trajectories in four analytic samples across sequential survey waves**

| **The first group between in the second and third surveys waves (n= 9,385)** | | | | | | | |
| --- | --- | --- | --- | --- | --- | --- | --- |
|  |  | **Model 1** | | | **Model 2** | | |
|  |  | **Improved to low risk (n= 283)** | **Increased to high risk (n= 354)** | **Persistent high risk (n= 136)** | **Improved to low risk (n= 283)** | **Increased to high risk (n= 354)** | **Persistent high risk (n= 136)** |
|  |  | **OR (95% CI)** | **OR (95% CI)** | **OR (95% CI)** | **OR (95% CI)** | **OR (95% CI)** | **OR (95% CI)** |
| **Age groups** | **Middle age (ref: young)** | 0.37 ( 0.24 - 0.58 ) | 0.24 ( 0.15 - 0.38 ) | 0.16 ( 0.06 - 0.41 ) | 0.54 ( 0.34 - 0.84 ) | 0.39 ( 0.24 - 0.63 ) | 0.30 ( 0.12 - 0.78 ) |
|  | **Elderly** | 0.68 ( 0.53 - 0.88 ) | 0.73 ( 0.58 - 0.92 ) | 0.81 ( 0.56 - 1.17 ) | 0.74 ( 0.57 - 0.96 ) | 0.83 ( 0.66 - 1.06 ) | 0.95 ( 0.65 - 1.38 ) |
| **Sex** | **Men (ref: women)** | 1.15 ( 0.89 - 1.48 ) | 1.08 ( 0.86 - 1.36 ) | 0.97 ( 0.67 - 1.40 ) | 1.08 ( 0.84 - 1.40 ) | 1.01 ( 0.80 - 1.28 ) | 0.86 ( 0.59 - 1.25 ) |
| **Occupation** | **Service industries (ref: government workers)** |  |  |  | 1.21 ( 0.85 - 1.73 ) | 1.00 ( 0.73 - 1.37 ) | 0.79 ( 0.50 - 1.26 ) |
|  | **Education sector** |  |  |  | 1.71 ( 1.13 - 2.58 ) | 2.00 ( 1.41 - 2.83 ) | 1.69 ( 1.01 - 2.81 ) |
|  | **All other** |  |  |  | 0.49 ( 0.33 - 0.74 ) | 0.39 ( 0.27 - 0.56 ) | 0.19 ( 0.10 - 0.36 ) |
| **Residential areas** | **Inland areas (ref: coastal and mountainous areas)** |  |  |  | 0.82 ( 0.61 - 1.09 ) | 0.78 ( 0.60 - 1.01 ) | 1.33 ( 0.83 - 2.13 ) |
| **Receiving feedback** | **Receive feedback (ref: no receive feedback)** | 0.98 ( 0.70 - 1.36 ) | 1.46 ( 1.04 - 2.06 ) | 0.73 ( 0.48 - 1.13 ) | 0.99 ( 0.71 - 1.39 ) | 1.50 ( 1.06 - 2.12 ) | 0.77 ( 0.50 - 1.19 ) |

| **The second group between in the third and fourth surveys waves (n= 11,907)** | | | | | | | |
| --- | --- | --- | --- | --- | --- | --- | --- |
|  |  | **Model 1** | | | **Model 2** | | |
|  |  | **Improved to low risk (n= 493)** | **Increased to high risk (n= 956)** | **Persistent high risk (n= 53)** | **Improved to low risk (n= 493)** | **Increased to high risk (n= 956)** | **Persistent high risk (n= 53)** |
|  |  | **OR (95% CI)** | **OR (95% CI)** | **OR (95% CI)** | **OR (95% CI)** | **OR (95% CI)** | **OR (95% CI)** |
| **Age groups** | **Middle age (ref: young)** | 0.19 ( 0.13 - 0.27 ) | 1.10 ( 0.89 - 1.34 ) | 0.17 ( 0.06 - 0.50 ) | 0.36 ( 0.24 - 0.53 ) | 1.12 ( 0.91 - 1.39 ) | 0.29 ( 0.09 - 0.89 ) |
|  | **Elderly** | 0.73 ( 0.59 - 0.90 ) | 0.97 ( 0.81 - 1.16 ) | 0.55 ( 0.31 - 1.00 ) | 0.89 ( 0.72 - 1.11 ) | 0.98 ( 0.82 - 1.18 ) | 0.65 ( 0.36 - 1.19 ) |
| **Sex** | **Men (ref: women)** | 1.09 ( 0.90 - 1.32 ) | 1.02 ( 0.88 - 1.17 ) | 0.64 ( 0.34 - 1.23 ) | 1.02 ( 0.83 - 1.24 ) | 1.01 ( 0.88 - 1.17 ) | 0.63 ( 0.33 - 1.21 ) |
| **Occupation** | **Service industries (ref: government workers)** |  |  |  | 0.94 ( 0.72 - 1.24 ) | 0.99 ( 0.79 - 1.25 ) | 1.74 ( 0.66 - 4.59 ) |
|  | **Education sector** |  |  |  | 2.18 ( 1.61 - 2.95 ) | 1.09 ( 0.80 - 1.47 ) | 3.23 ( 1.13 - 9.18 ) |
|  | **All other** |  |  |  | 0.31 ( 0.22 - 0.43 ) | 0.97 ( 0.77 - 1.22 ) | 0.61 ( 0.20 - 1.82 ) |
| **Residential areas** | **Inland areas (ref: coastal and mountainous areas)** |  |  |  | 1.05 ( 0.83 - 1.32 ) | 1.13 ( 0.95 - 1.35 ) | 0.47 ( 0.26 - 0.83 ) |
| **Receiving feedback** | **Receive feedback (ref: no receive feedback)** | 1.32 ( 0.99 - 1.75 ) | 0.93 ( 0.77 - 1.12 ) | 1.47 ( 0.62 - 3.48 ) | 1.30 ( 0.97 - 1.72 ) | 0.93 ( 0.77 - 1.12 ) | 1.49 ( 0.63 - 3.51 ) |

| **The third group between in the fourth and fifth surveys waves (n= 9,635)** | | | | | | | |
| --- | --- | --- | --- | --- | --- | --- | --- |
|  |  | **Model 1** | | | **Model 2** | | |
|  |  | **Improved to low risk (n= 614)** | **Increased to high risk (n= 104)** | **Persistent high risk (n= 9)** | **Improved to low risk (n= 614)** | **Increased to high risk (n= 104)** | **Persistent high risk (n= 9)** |
|  |  | **OR (95% CI)** | **OR (95% CI)** | **OR (95% CI)** | **OR (95% CI)** | **OR (95% CI)** | **OR (95% CI)** |
| **Age groups** | **Middle age and elderly (ref: young)** | 0.56 ( 0.47 - 0.68 ) | 1.52 ( 0.87 - 2.64 ) | 0.23 ( 0.06 - 0.86 ) | 0.74 ( 0.61 - 0.89 ) | 1.39 ( 0.79 - 2.44 ) | 0.30 ( 0.07 - 1.20 ) |
| **Sex** | **Men (ref: women)** | 0.95 ( 0.80 - 1.13 ) | 0.96 ( 0.64 - 1.44 ) | 1.09 ( 0.27 - 4.39 ) | 0.87 ( 0.73 - 1.04 ) | 0.98 ( 0.65 - 1.43 ) | 0.96 ( 0.23 - 3.94 ) |
| **Occupation** | **Service industries (ref: government workers)** |  |  |  | 0.81 ( 0.63 - 1.03 ) | 1.91 ( 0.85 - 4.26 ) | 0.72 ( 0.13 - 4.07 ) |
|  | **Education sector** |  |  |  | 1.48 ( 1.12 - 1.97 ) | 0.78 ( 0.23 - 2.69 ) | 1.04 ( 0.14 - 7.70 ) |
|  | **All other** |  |  |  | 0.28 ( 0.21 - 0.37 ) | 1.65 ( 0.74 - 3.67 ) | 0.16 ( 0.01 - 1.81 ) |
| **Residential areas** | **Inland areas (ref: coastal and mountainous areas)** |  |  |  | 1.09 ( 0.88 - 1.36 ) | 1.10 ( 0.67 - 1.82 ) | 0.50 ( 0.12 - 2.00 ) |
| **Receiving feedback** | **Receive feedback (ref: no receive feedback)** | 1.31 ( 1.04 - 1.63 ) | 1.20 ( 0.70 - 2.05 ) | 0.61 ( 0.15 - 2.53 ) | 1.29 ( 1.03 - 1.62 ) | 1.19 ( 0.69 - 2.05 ) | 0.61 ( 0.15 - 2.50 ) |

| **The fourth group between in the fifth and sixth surveys waves (n= 10,636)** | | | | | | | |
| --- | --- | --- | --- | --- | --- | --- | --- |
|  |  | **Model 1** | | | **Model 2** | | |
|  |  | **Improved to low risk (n= 41)** | **Increased to high risk (n= 1192)** | **Persistent high risk (n= 51)** | **Improved to low risk (n= 41)** | **Increased to high risk (n= 1192)** | **Persistent high risk (n= 51)** |
|  |  | **OR (95% CI)** | **OR (95% CI)** | **OR (95% CI)** | **OR (95% CI)** | **OR (95% CI)** | **OR (95% CI)** |
| **Age groups** | **Middle age (ref: young)** | 0.20 ( 0.06 - 0.62 ) | 0.28 ( 0.22 - 0.34 ) | 0.14 ( 0.05 - 0.38 ) | 0.40 ( 0.12 - 1.31 ) | 0.34 ( 0.28 - 0.42 ) | 0.15 ( 0.05 - 0.42 ) |
|  | **Elderly** | 0.54 ( 0.27 - 1.06 ) | 0.62 ( 0.54 - 0.72 ) | 0.39 ( 0.21 - 0.70 ) | 0.67 ( 0.34 - 1.34 ) | 0.64 ( 0.56 - 0.74 ) | 0.39 ( 0.21 - 0.71 ) |
| **Sex** | **Men (ref: women)** | 1.36 ( 0.72 - 2.55 ) | 0.92 ( 0.80 - 1.05 ) | 1.89 ( 1.09 - 3.29 ) | 1.23 ( 0.65 - 2.32 ) | 0.86 ( 0.75 - 0.98 ) | 1.90 ( 1.09 - 3.32 ) |
| **Occupation** | **Service industries (ref: government workers)** |  |  |  | 0.75 ( 0.30 - 1.87 ) | 0.87 ( 0.72 - 1.04 ) | 1.70 ( 0.64 - 4.54 ) |
|  | **Education sector** |  |  |  | 2.21 ( 0.88 - 5.57 ) | 0.92 ( 0.73 - 1.16 ) | 1.76 ( 0.57 - 5.45 ) |
|  | **All other** |  |  |  | 0.27 ( 0.09 - 0.82 ) | 0.54 ( 0.44 - 0.65 ) | 1.27 ( 0.46 - 3.51 ) |
| **Residential areas** | **Inland areas (ref: coastal and mountainous areas)** |  |  |  | 0.82 ( 0.40 - 1.69 ) | 1.13 ( 0.97 - 1.31 ) | 1.26 ( 0.61 - 2.59 ) |
| **Receiving feedback** | **Receive feedback (ref: no receive feedback)** | 0.49 ( 0.25 - 0.96 ) | 1.09 ( 0.92 - 1.28 ) | 1.81 ( 0.77 - 4.30 ) | 0.50 ( 0.26 - 0.98 ) | 1.10 ( 0.93 - 1.29 ) | 1.84 ( 0.78 - 4.36 ) |

Abbreviations: CI, confidence interval; OR, odds ratio.

**Supplementary Table 3. Results of analysis for risk trajectories by multiple imputation methods in four analytic samples across sequential survey waves**

| **The first group between in the second and third surveys waves (n= 9,385)** | | | | | | | |
| --- | --- | --- | --- | --- | --- | --- | --- |
|  |  | **Model 1** | | | **Model 2** | | |
|  |  | **Improved to low risk (n= 283)** | **Increased to high risk (n= 354)** | **Persistent high risk (n= 136)** | **Improved to low risk (n= 283)** | **Increased to high risk (n= 354)** | **Persistent high risk (n= 136)** |
|  |  | **OR (95% CI)** | **OR (95% CI)** | **OR (95% CI)** | **OR (95% CI)** | **OR (95% CI)** | **OR (95% CI)** |
| **Age groups** | **Middle age (ref: young)** | 0.68 ( 0.60 - 0.78 ) | 0.73 ( 0.65 - 0.82 ) | 0.82 ( 0.68 - 0.98 ) | 0.74 ( 0.65 - 0.85 ) | 0.83 ( 0.74 - 0.94 ) | 0.95 ( 0.79 - 1.15 ) |
|  | **Elderly** | 0.38 ( 0.30 - 0.47 ) | 0.24 ( 0.15 - 0.38 ) | 0.16 ( 0.10 - 0.26 ) | 0.54 ( 0.43 - 0.68 ) | 0.39 ( 0.24 - 0.63 ) | 0.31 ( 0.19 - 0.50 ) |
| **Sex** | **Men (ref: women)** | 1.15 ( 0.89 - 1.47 ) | 1.08 ( 0.86 - 1.36 ) | 0.97 ( 0.67 - 1.40 ) | 1.08 ( 0.84 - 1.40 ) | 1.01 ( 0.80 - 1.27 ) | 0.86 ( 0.59 - 1.25 ) |
| **Occupation** | **Service industries (ref: government workers)** |  |  |  | 1.21 ( 1.01 - 1.45 ) | 0.98 ( 0.72 - 1.34 ) | 0.79 ( 0.62 - 1.00 ) |
|  | **Education sector** |  |  |  | 1.70 ( 1.37 - 2.10 ) | 1.95 ( 1.38 - 2.75 ) | 1.68 ( 1.29 - 2.18 ) |
|  | **All other** |  |  |  | 0.49 ( 0.40 - 0.60 ) | 0.38 ( 0.26 - 0.55 ) | 0.19 ( 0.13 - 0.26 ) |
| **Residential areas** | **Inland areas (ref: coastal and mountainous areas)** |  |  |  | 0.82 ( 0.71 - 0.95 ) | 0.79 ( 0.69 - 0.90 ) | 1.33 ( 1.05 - 1.69 ) |
| **Receiving feedback** | **Receive feedback (ref: no receive feedback)** | 0.97 ( 0.82 - 1.15 ) | 1.47 ( 1.23 - 1.76 ) | 0.73 ( 0.59 - 0.91 ) | 0.99 ( 0.84 - 1.18 ) | 1.51 ( 1.27 - 1.80 ) | 0.77 ( 0.62 - 0.96 ) |

| **The second group between in the third and fourth surveys waves (n= 11,907)** | | | | | | | |
| --- | --- | --- | --- | --- | --- | --- | --- |
|  |  | **Model 1** | | | **Model 2** | | |
|  |  | **Improved to low risk (n= 493)** | **Increased to high risk (n= 956)** | **Persistent high risk (n= 53)** | **Improved to low risk (n= 493)** | **Increased to high risk (n= 956)** | **Persistent high risk (n= 53)** |
|  |  | **OR (95% CI)** | **OR (95% CI)** | **OR (95% CI)** | **OR (95% CI)** | **OR (95% CI)** | **OR (95% CI)** |
| **Age groups** | **Middle age (ref: young)** | 0.74 ( 0.60 - 0.91 ) | 0.97 ( 0.89 - 1.06 ) | 0.56 ( 0.41 - 0.75 ) | 0.91 ( 0.74 - 1.12 ) | 0.98 ( 0.83 - 1.16 ) | 0.65 ( 0.48 - 0.89 ) |
|  | **Elderly** | 0.19 ( 0.13 - 0.28 ) | 1.10 ( 0.90 - 1.34 ) | 0.17 ( 0.10 - 0.30 ) | 0.37 ( 0.25 - 0.55 ) | 1.12 ( 0.91 - 1.39 ) | 0.29 ( 0.16 - 0.51 ) |
| **Sex** | **Men (ref: women)** | 1.10 ( 0.90 - 1.33 ) | 1.02 ( 0.88 - 1.17 ) | 0.64 ( 0.46 - 0.89 ) | 1.02 ( 0.84 - 1.24 ) | 1.01 ( 0.88 - 1.17 ) | 0.63 ( 0.45 - 0.88 ) |
| **Occupation** | **Service industries (ref: government workers)** |  |  |  | 0.93 ( 0.71 - 1.22 ) | 1.00 ( 0.79 - 1.26 ) | 1.73 ( 1.05 - 2.84 ) |
|  | **Education sector** |  |  |  | 2.20 ( 1.63 - 2.98 ) | 1.08 ( 0.80 - 1.46 ) | 3.21 ( 1.89 - 5.48 ) |
|  | **All other** |  |  |  | 0.30 ( 0.21 - 0.42 ) | 0.98 ( 0.78 - 1.23 ) | 0.61 ( 0.35 - 1.06 ) |
| **Residential areas** | **Inland areas (ref: coastal and mountainous areas)** |  |  |  | 1.06 ( 0.94 - 1.20 ) | 1.13 ( 1.04 - 1.23 ) | 0.47 ( 0.26 - 0.83 ) |
| **Receiving feedback** | **Receive feedback (ref: no receive feedback)** | 1.33 ( 1.15 - 1.54 ) | 0.93 ( 0.85 - 1.03 ) | 1.48 ( 0.95 - 2.29 ) | 1.31 ( 1.13 - 1.52 ) | 0.93 ( 0.84 - 1.02 ) | 1.49 ( 0.96 - 2.32 ) |

| **The third group between in the fourth and fifth surveys waves (n= 9,635)** | | | | | | | |
| --- | --- | --- | --- | --- | --- | --- | --- |
|  |  | **Model 1** | | | **Model 2** | | |
|  |  | **Improved to low risk (n= 614)** | **Increased to high risk (n= 104)** | **Persistent high risk (n= 9)** | **Improved to low risk (n= 614)** | **Increased to high risk (n= 104)** | **Persistent high risk (n= 9)** |
|  |  | **OR (95% CI)** | **OR (95% CI)** | **OR (95% CI)** | **OR (95% CI)** | **OR (95% CI)** | **OR (95% CI)** |
| **Age groups** | **Middle age and elderly (ref: young)** | 0.57 ( 0.47 - 0.68 ) | 1.52 ( 1.14 - 2.03 ) | 0.23 ( 0.11 - 0.45 ) | 0.74 ( 0.61 - 0.90 ) | 1.39 ( 1.04 - 1.85 ) | 0.30 ( 0.14 - 0.62 ) |
| **Sex** | **Men (ref: women)** | 0.95 ( 0.80 - 1.13 ) | 0.95 ( 0.64 - 1.43 ) | 1.09 ( 0.31 - 3.87 ) | 0.87 ( 0.73 - 1.04 ) | 0.98 ( 0.65 - 1.47 ) | 0.96 ( 0.47 - 1.97 ) |
| **Occupation** | **Service industries (ref: government workers)** |  |  |  | 0.80 ( 0.63 - 1.01 ) | 1.90 ( 1.26 - 2.87 ) | 0.73 ( 0.30 - 1.77 ) |
|  | **Education sector** |  |  |  | 1.46 ( 1.10 - 1.93 ) | 0.78 ( 0.41 - 1.48 ) | 1.05 ( 0.37 - 2.97 ) |
|  | **All other** |  |  |  | 0.28 ( 0.21 - 0.37 ) | 1.64 ( 1.09 - 2.48 ) | 0.16 ( 0.05 - 0.54 ) |
| **Residential areas** | **Inland areas (ref: coastal and mountainous areas)** |  |  |  | 1.09 ( 0.98 - 1.22 ) | 1.10 ( 0.75 - 1.62 ) | 0.50 ( 0.50 - 0.50 ) |
| **Receiving feedback** | **Receive feedback (ref: no receive feedback)** | 1.29 ( 1.15 - 1.45 ) | 1.20 ( 1.20 - 1.20 ) | 0.62 ( 0.30 - 1.28 ) | 1.28 ( 1.14 - 1.43 ) | 1.20 ( 1.20 - 1.20 ) | 0.61 ( 0.61 - 0.61 ) |

| **The fourth group between in the fifth and sixth surveys waves (n= 10,636)** | | | | | | | |
| --- | --- | --- | --- | --- | --- | --- | --- |
|  |  | **Model 1** | | | **Model 2** | | |
|  |  | **Improved to low risk (n= 41)** | **Increased to high risk (n= 1192)** | **Persistent high risk (n= 51)** | **Improved to low risk (n= 41)** | **Increased to high risk (n= 1192)** | **Persistent high risk (n= 51)** |
|  |  | **OR (95% CI)** | **OR (95% CI)** | **OR (95% CI)** | **OR (95% CI)** | **OR (95% CI)** | **OR (95% CI)** |
| **Age groups** | **Middle age (ref: young)** | 0.54 ( 0.38 - 0.77 ) | 0.62 ( 0.58 - 0.67 ) | 0.39 ( 0.29 - 0.53 ) | 0.68 ( 0.47 - 0.96 ) | 0.64 ( 0.60 - 0.69 ) | 0.39 ( 0.29 - 0.53 ) |
|  | **Elderly** | 0.20 ( 0.11 - 0.36 ) | 0.28 ( 0.23 - 0.34 ) | 0.14 ( 0.09 - 0.24 ) | 0.41 ( 0.22 - 0.74 ) | 0.34 ( 0.28 - 0.42 ) | 0.15 ( 0.09 - 0.26 ) |
| **Sex** | **Men (ref: women)** | 1.36 ( 0.72 - 2.56 ) | 0.92 ( 0.81 - 1.05 ) | 1.89 ( 1.09 - 3.29 ) | 1.22 ( 0.88 - 1.70 ) | 0.86 ( 0.75 - 0.99 ) | 1.90 ( 1.09 - 3.30 ) |
| **Occupation** | **Service industries (ref: government workers)** |  |  |  | 0.75 ( 0.47 - 1.19 ) | 0.85 ( 0.71 - 1.02 ) | 1.69 ( 1.02 - 2.79 ) |
|  | **Education sector** |  |  |  | 2.20 ( 1.37 - 3.53 ) | 0.90 ( 0.75 - 1.09 ) | 1.75 ( 0.98 - 3.11 ) |
|  | **All other** |  |  |  | 0.27 ( 0.15 - 0.47 ) | 0.52 ( 0.43 - 0.64 ) | 1.26 ( 0.75 - 2.12 ) |
| **Residential areas** | **Inland areas (ref: coastal and mountainous areas)** |  |  |  | 0.82 ( 0.57 - 1.19 ) | 1.13 ( 1.04 - 1.22 ) | 1.25 ( 0.85 - 1.85 ) |
| **Receiving feedback** | **Receive feedback (ref: no receive feedback)** | 0.49 ( 0.35 - 0.70 ) | 1.09 ( 1.00 - 1.19 ) | 1.82 ( 1.17 - 2.82 ) | 0.50 ( 0.36 - 0.71 ) | 1.10 ( 1.01 - 1.20 ) | 1.85 ( 1.19 - 2.87 ) |

Abbreviations: CI, confidence interval; OR, odds ratio.

**Supplementary Table 4. Comparison of demographic characteristics between those who participated in the prior survey but did not participated in the next survey and those who participated in the both survey waves**

| **The first group between in the second and third surveys waves (n=15,017)** | | | | | |
| --- | --- | --- | --- | --- | --- |
|  |  | **Missing** | **Non participants (n=5,632)** | **Participants (n=9,385)** |  |
|  |  | **n (%)** | **n (%)** | **n (%)** | ***P* value** |
| **Age groups** | **Young** | 0 (0.0) | 2389 (42.4) | 2437 (26.0) | < 0.001 |
|  | **Middle age** |  | 2759 (49.0) | 5374 (57.3) |  |
|  | **Elderly** |  | 484 (8.6) | 1574 (16.8) |  |
| **Sex** | **Men** | 69 (0.5) | 2149 (38.4) | 3117 (33.4) | < 0.001 |
| **Occupation** | **Government workers** | 0 (0.0) | 2286 (40.6) | 3364 (35.8) | < 0.001 |
|  | **Service industries** |  | 724 (12.9) | 1007 (10.7) |  |
|  | **Education sector** |  | 1732 (30.8) | 3725 (39.7) |  |
|  | **All other** |  | 890 (15.8) | 1289 (13.7) |  |
| **Residential areas** | **Inland areas** | 0 (0.0) | 4507 (80.0) | 7576 (80.7) | 0.295 |
| **Receive feedback** | **Receiving feedback** | 0 (0.0) | 4437 (78.8) | 8091 (86.2) | < 0.001 |

| **The second group between in the third and fourth surveys waves (n=23,962)** | | | | | |
| --- | --- | --- | --- | --- | --- |
|  |  | **Missing** | **Non participants (n=12,055)** | **Participants (n=11,907)** |  |
|  |  | **n (%)** | **n (%)** | **n (%)** | ***P* value** |
| **Age groups** | **Young** | 0 (0.0) | 3822 (31.7) | 2262 (19.0) | < 0.001 |
|  | **Middle age** |  | 6312 (52.4) | 6776 (56.9) |  |
|  | **Elderly** |  | 1921 (15.9) | 2869 (24.1) |  |
| **Sex** | **Men** | 152 (0.6) | 4388 (36.6) | 3999 (33.8) | < 0.001 |
| **Occupation** | **Government workers** | 0 (0.0) | 4920 (40.8) | 4261 (35.8) | < 0.001 |
|  | **Service industries** |  | 1145 (9.5) | 1091 (9.2) |  |
|  | **Education sector** |  | 4690 (38.9) | 5213 (43.8) |  |
|  | **All other** |  | 1300 (10.8) | 1342 (11.3) |  |
| **Residential areas** | **Inland areas** | 0 (0.0) | 9573 (79.4) | 9526 (80.0) | 0.254 |
| **Receive feedback** | **Receiving feedback** | 0 (0.0) | 9479 (78.6) | 10284 (86.4) | < 0.001 |

| **The third group between in the fourth and fifth surveys waves (n=19,188)** | | | | | |
| --- | --- | --- | --- | --- | --- |
|  |  | **Missing** | **Non participants (n=9553)** | **Participants (n=9635)** |  |
|  |  | **n (%)** | **n (%)** | **n (%)** | ***P* value** |
| **Age groups** | **Young** | 0 (0.0) | 2046 (21.4) | 2059 (21.4) | 0.936 |
|  | **Middle age and elderly** |  | 7507 (78.6) | 7576 (78.6) |  |
| **Sex** | **Men** | 115 (0.6) | 3424 (36.1) | 3348 (34.9) | 0.091 |
| **Occupation** | **Government workers** | 0 (0.0) | 3472 (36.3) | 3549 (36.8) | 0.825 |
|  | **Service industries** |  | 912 (9.5) | 892 (9.3) |  |
|  | **Education sector** |  | 4092 (42.8) | 4097 (42.5) |  |
|  | **All other** |  | 1077 (11.3) | 1097 (11.4) |  |
| **Residential areas** | **Inland areas** | 0 (0.0) | 7745 (81.1) | 7717 (80.1) | 0.086 |
| **Receive feedback** | **Receiving feedback** | 0 (0.0) | 7758 (81.2) | 7838 (81.3) | 0.805 |

| **The fourth group between in the fifth and sixth surveys waves (n=18,444)** | | | | | |
| --- | --- | --- | --- | --- | --- |
|  |  | **Missing** | **Non participants (n=9553)** | **Participants (n=9635)** |  |
|  |  | **n (%)** | **n (%)** | **n (%)** | ***P* value** |
| **Age groups** | **Young** | 0 (0.0) | 2122 (27.2) | 1962 (18.4) | < 0.001 |
|  | **Middle age** |  | 4317 (55.3) | 6035 (56.7) |  |
|  | **Elderly** |  | 1369 (17.5) | 2639 (24.8) |  |
| **Sex** | **Men** | 110 (0.6) | 2781 (35.9) | 3622 (34.2) | 0.021 |
| **Occupation** | **Government workers** | 0 (0.0) | 3128 (40.1) | 3603 (33.9) | < 0.001 |
|  | **Service industries** |  | 687 (8.8) | 1094 (10.3) |  |
|  | **Education sector** |  | 3183 (40.8) | 4666 (43.9) |  |
|  | **All other** |  | 810 (10.4) | 1273 (12.0) |  |
| **Residential areas** | **Inland areas** | 0 (0.0) | 6214 (79.6) | 8414 (79.1) | 0.086 |
| **Receive feedback** | **Receiving feedback** | 0 (0.0) | 5842 (74.8) | 8901 (83.7) | < 0.001 |

Categorical variables are presented as number of cases (%).

P values were calculated using the chi-squared test or Fisher’s exact test.

**Supplementary Table 5. Results of analysis for risk trajectories using inverse propensity weighting in four analytic samples across sequential survey waves**

| **The first group between in the second and third surveys waves (n= 9,346)** | | | | | | | |
| --- | --- | --- | --- | --- | --- | --- | --- |
|  |  | **Model 1** | | | **Model 2** | | |
|  |  | **Improved to low risk (n= 283)** | **Increased to high risk (n= 352)** | **Persistent high risk (n= 136)** | **Improved to low risk (n= 283)** | **Increased to high risk (n= 352)** | **Persistent high risk (n= 136)** |
|  |  | **OR (95% CI)** | **OR (95% CI)** | **OR (95% CI)** | **OR (95% CI)** | **OR (95% CI)** | **OR (95% CI)** |
| **Age groups** | **Middle age (ref: young)** | 0.68 ( 0.56 - 0.83 ) | 0.74 ( 0.63 - 0.88 ) | 0.80 ( 0.61 - 1.04 ) | 0.74 ( 0.61 - 0.90 ) | 0.85 ( 0.71 - 1.01 ) | 0.92 ( 0.70 - 1.21 ) |
|  | **Elderly** | 0.38 ( 0.26 - 0.55 ) | 0.25 ( 0.17 - 0.37 ) | 0.16 ( 0.07 - 0.35 ) | 0.55 ( 0.38 - 0.80 ) | 0.40 ( 0.27 - 0.60 ) | 0.30 ( 0.13 - 0.66 ) |
| **Sex** | **Men (ref: women)** | 1.12 ( 0.93 - 1.36 ) | 1.02 ( 0.86 - 1.22 ) | 0.97 ( 0.74 - 1.28 ) | 1.06 ( 0.87 - 1.29 ) | 0.96 ( 0.81 - 1.15 ) | 0.86 ( 0.65 - 1.14 ) |
| **Occupation** | **Service industries (ref: government workers)** |  |  |  | 1.16 ( 0.89 - 1.51 ) | 1.02 ( 0.80 - 1.30 ) | 0.75 ( 0.53 - 1.06 ) |
|  | **Education sector** |  |  |  | 1.63 ( 1.19 - 2.23 ) | 2.02 ( 1.54 - 2.64 ) | 1.50 ( 1.02 - 2.21 ) |
|  | **All other** |  |  |  | 0.47 ( 0.34 - 0.65 ) | 0.41 ( 0.30 - 0.54 ) | 0.18 ( 0.11 - 0.30 ) |
| **Residential areas** | **Inland areas (ref: coastal and mountainous areas)** |  |  |  | 0.83 ( 0.67 - 1.04 ) | 0.78 ( 0.64 - 0.95 ) | 1.42 ( 0.98 - 2.06 ) |
| **Receiving feedback** | **Receive feedback (ref: no receive feedback)** | 0.92 ( 0.72 - 1.18 ) | 1.49 ( 1.15 - 1.94 ) | 0.77 ( 0.56 - 1.08 ) | 0.94 ( 0.74 - 1.21 ) | 1.53 ( 1.18 - 1.99 ) | 0.81 ( 0.58 - 1.13 ) |

| **The second group between in the third and fourth surveys waves (n= 11,836)** | | | | | | | |
| --- | --- | --- | --- | --- | --- | --- | --- |
|  |  | **Model 1** | | | **Model 2** | | |
|  |  | **Improved to low risk (n= 488)** | **Increased to high risk (n= 949)** | **Persistent high risk (n= 53)** | **Improved to low risk (n= 488)** | **Increased to high risk (n= 949)** | **Persistent high risk (n= 53)** |
|  |  | **OR (95% CI)** | **OR (95% CI)** | **OR (95% CI)** | **OR (95% CI)** | **OR (95% CI)** | **OR (95% CI)** |
| **Age groups** | **Middle age (ref: young)** | 0.81 ( 0.66 - 1.00 ) | 1.12 ( 0.93 - 1.36 ) | 0.58 ( 0.33 - 1.03 ) | 0.97 ( 0.78 - 1.20 ) | 1.15 ( 0.95 - 1.39 ) | 0.69 ( 0.38 - 1.23 ) |
|  | **Elderly** | 0.27 ( 0.18 - 0.40 ) | 1.39 ( 1.10 - 1.75 ) | NA | 0.53 ( 0.34 - 0.81 ) | 1.40 ( 1.10 - 1.79 ) | NA |
| **Sex** | **Men (ref: women)** | 1.01 ( 0.82 - 1.24 ) | 1.15 ( 0.98 - 1.34 ) | 0.62 ( 0.31 - 1.22 ) | 0.94 ( 0.76 - 1.16 ) | 1.16 ( 0.99 - 1.36 ) | 0.66 ( 0.33 - 1.31 ) |
| **Occupation** | **Service industries (ref: government workers)** |  |  |  | 0.94 ( 0.71 - 1.23 ) | 1.07 ( 0.83 - 1.38 ) | 3.04 ( 0.90 - 10.24 ) |
|  | **Education sector** |  |  |  | 2.29 ( 1.69 - 3.10 ) | 1.44 ( 1.04 - 1.98 ) | 6.87 ( 1.97 - 24.01 ) |
|  | **All other** |  |  |  | 0.25 ( 0.18 - 0.36 ) | 1.13 ( 0.88 - 1.46 ) | 1.38 ( 0.37 - 5.12 ) |
| **Residential areas** | **Inland areas (ref: coastal and mountainous areas)** |  |  |  | 0.87 ( 0.69 - 1.11 ) | 1.07 ( 0.88 - 1.30 ) | 0.50 ( 0.28 - 0.92 ) |
| **Receiving feedback** | **Receive feedback (ref: no receive feedback)** | 1.37 ( 0.99 - 1.89 ) | 1.19 ( 0.93 - 1.54 ) | 0.77 ( 0.37 - 1.63 ) | 1.38 ( 0.99 - 1.92 ) | 1.20 ( 0.93 - 1.55 ) | 0.82 ( 0.38 - 1.73 ) |

| **The third group between in the fourth and fifth surveys waves (n= 9,587)** | | | | | | | |
| --- | --- | --- | --- | --- | --- | --- | --- |
|  |  | **Model 1** | | | **Model 2** | | |
|  |  | **Improved to low risk (n= 610)** | **Increased to high risk (n= 104)** | **Persistent high risk (n= 9)** | **Improved to low risk (n= 610)** | **Increased to high risk (n= 104)** | **Persistent high risk (n= 9)** |
|  |  | **OR (95% CI)** | **OR (95% CI)** | **OR (95% CI)** | **OR (95% CI)** | **OR (95% CI)** | **OR (95% CI)** |
| **Age groups** | **Middle age and elderly (ref: young)** | 0.55 ( 0.46 - 0.66 ) | 1.39 ( 0.73 - 2.64 ) | 0.31 ( 0.09 - 1.15 ) | 0.74 ( 0.61 - 0.90 ) | 1.36 ( 0.71 - 2.61 ) | 0.38 ( 0.10 - 1.45 ) |
| **Sex** | **Men (ref: women)** | 0.93 ( 0.77 - 1.11 ) | 0.89 ( 0.55 - 1.45 ) | 1.12 ( 0.29 - 4.32 ) | 0.83 ( 0.70 - 1.00 ) | 0.87 ( 0.53 - 1.43 ) | 1.04 ( 0.27 - 4.05 ) |
| **Occupation** | **Service industries (ref: government workers)** |  |  |  | 0.72 ( 0.56 - 0.91 ) | 1.64 ( 0.70 - 3.83 ) | 1.13 ( 0.18 - 7.07 ) |
|  | **Education sector** |  |  |  | 1.33 ( 1.00 - 1.76 ) | 0.65 ( 0.17 - 2.50 ) | 1.05 ( 0.11 - 9.95 ) |
|  | **All other** |  |  |  | 0.22 ( 0.16 - 0.29 ) | 0.93 ( 0.39 - 2.22 ) | 0.32 ( 0.03 - 2.99 ) |
| **Residential areas** | **Inland areas (ref: coastal and mountainous areas)** |  |  |  | 1.30 ( 1.04 - 1.64 ) | 1.16 ( 0.63 - 2.14 ) | 0.51 ( 0.13 - 2.00 ) |
| **Receiving feedback** | **Receive feedback (ref: no receive feedback)** | 1.54 ( 1.21 - 1.96 ) | 0.85 ( 0.47 - 1.52 ) | 0.27 ( 0.07 - 0.98 ) | 1.49 ( 1.17 - 1.90 ) | 0.84 ( 0.47 - 1.50 ) | 0.26 ( 0.07 - 0.95 ) |

| **The fourth group between in the fifth and sixth surveys waves (n= 10,581)** | | | | | | | |
| --- | --- | --- | --- | --- | --- | --- | --- |
|  |  | **Model 1** | | | **Model 2** | | |
|  |  | **Improved to low risk (n= 41)** | **Increased to high risk (n= 1184)** | **Persistent high risk (n= 51)** | **Improved to low risk (n= 41)** | **Increased to high risk (n= 1184)** | **Persistent high risk (n= 51)** |
|  |  | **OR (95% CI)** | **OR (95% CI)** | **OR (95% CI)** | **OR (95% CI)** | **OR (95% CI)** | **OR (95% CI)** |
| **Age groups** | **Middle age (ref: young)** | 0.37 ( 0.17 - 0.80 ) | 0.65 ( 0.55 - 0.77 ) | 0.29 ( 0.15 - 0.55 ) | 0.43 ( 0.19 - 0.94 ) | 0.67 ( 0.57 - 0.79 ) | 0.30 ( 0.16 - 0.59 ) |
|  | **Elderly** | 0.21 ( 0.06 - 0.66 ) | 0.25 ( 0.19 - 0.32 ) | 0.08 ( 0.02 - 0.30 ) | 0.37 ( 0.11 - 1.26 ) | 0.31 ( 0.24 - 0.40 ) | 0.10 ( 0.03 - 0.39 ) |
| **Sex** | **Men (ref: women)** | 1.01 ( 0.46 - 2.20 ) | 0.94 ( 0.81 - 1.09 ) | 1.14 ( 0.58 - 2.22 ) | 0.94 ( 0.43 - 2.06 ) | 0.89 ( 0.76 - 1.04 ) | 1.11 ( 0.57 - 2.17 ) |
| **Occupation** | **Service industries (ref: government workers)** |  |  |  | 0.77 ( 0.27 - 2.19 ) | 0.79 ( 0.64 - 0.97 ) | 2.29 ( 0.63 - 8.28 ) |
|  | **Education sector** |  |  |  | 1.64 ( 0.53 - 5.06 ) | 0.85 ( 0.65 - 1.11 ) | 3.58 ( 0.91 - 14.03 ) |
|  | **All other** |  |  |  | 0.28 ( 0.08 - 0.97 ) | 0.50 ( 0.40 - 0.62 ) | 1.26 ( 0.32 - 4.85 ) |
| **Residential areas** | **Inland areas (ref: coastal and mountainous areas)** |  |  |  | 0.87 ( 0.37 - 2.06 ) | 0.98 ( 0.82 - 1.17 ) | 2.13 ( 0.78 - 5.85 ) |
| **Receiving feedback** | **Receive feedback (ref: no receive feedback)** | 0.90 ( 0.35 - 2.28 ) | 1.19 ( 0.97 - 1.46 ) | 1.78 ( 0.65 - 4.88 ) | 0.91 ( 0.36 - 2.31 ) | 1.19 ( 0.97 - 1.46 ) | 1.90 ( 0.69 - 5.20 ) |

Abbreviations: CI, confidence interval; OR, odds ratio; NA, not available.

**Supplementary Table 6. Comparison of characteristics of the whole population in the 2022 local census in Iwate and the analytic sample in the last group (in the fifth to sixth survey waves)**

|  |  | **2022 local census** | **Analytic sample in the last group (in the fifth to sixth survey waves)** |
| --- | --- | --- | --- |
|  |  | **n (%)** | **n (%)** |
| **Sex** | **Men** | 560,300 (48.1) | 3,622 (34.2) |
|  | **Women** | 605,300 (51.9) | 6,959 (65.8) |
| **Age classes** | **Young** | 371,736 (31.9) | 1,962 (22.0) |
|  | **Middle age** | 306,288 (26.3) | 6,035 (60.2) |
|  | **Elderly** | 487,576 (41.8) | 2,639 (17.8) |
| **Area** | **Inland areas** | 897,608 (77.0) | 8,414 (80.6) |
|  | **Coastal and mountainous areas** | 267,992 (23.0) | 2,222 (19.4) |
