# Supplementary figures and images for: Individual feedback on risk for acquiring SARS-CoV-2 infection failed to change future risk behaviors during the COVID-19 pandemic in Japan
Source: SSM Popul Health. 2026 May 11;34:101931. doi: 10.1016/j.ssmph.2026.101931 (PMC13199801; doi:10.1016/j.ssmph.2026.101931)

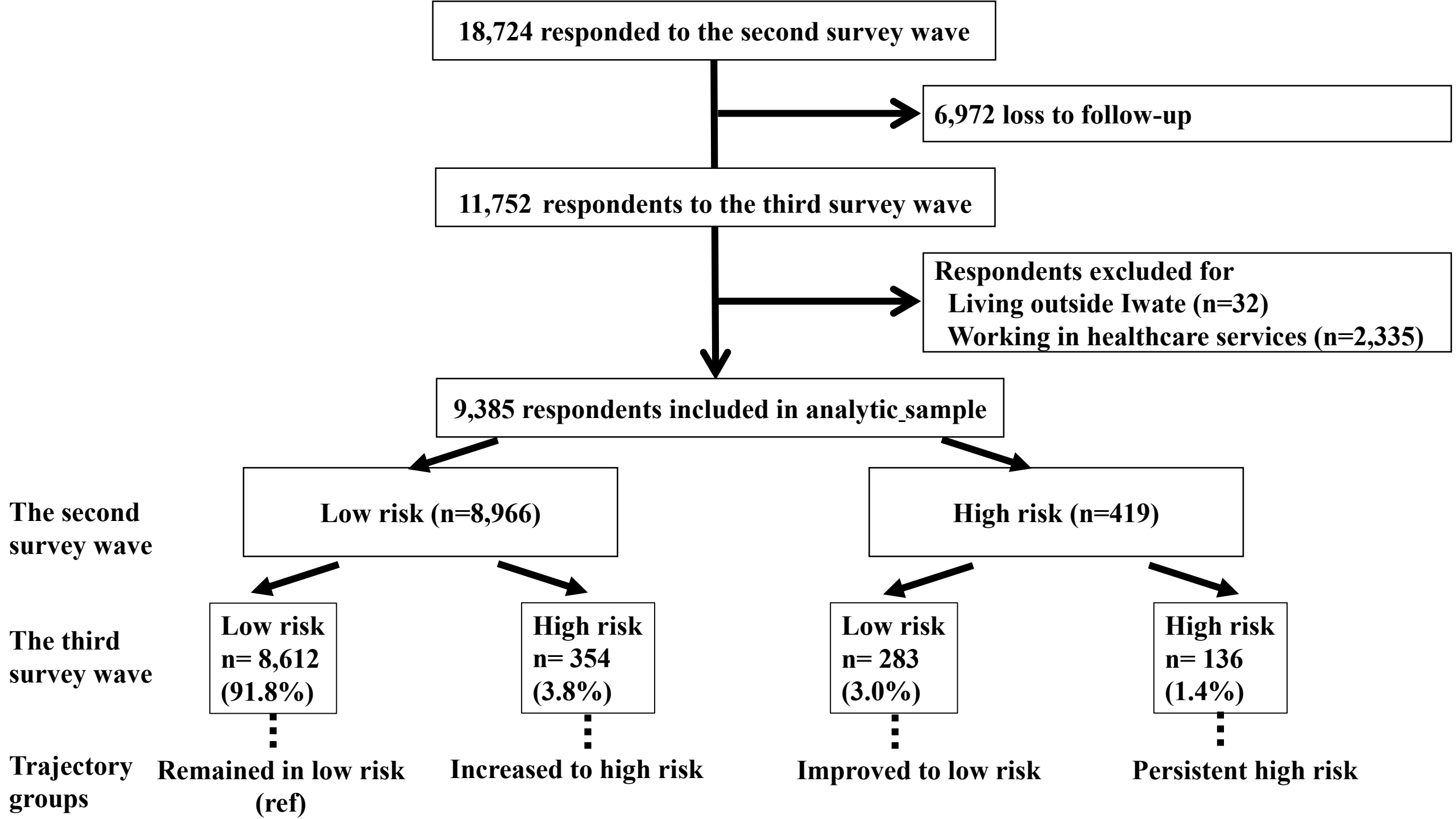

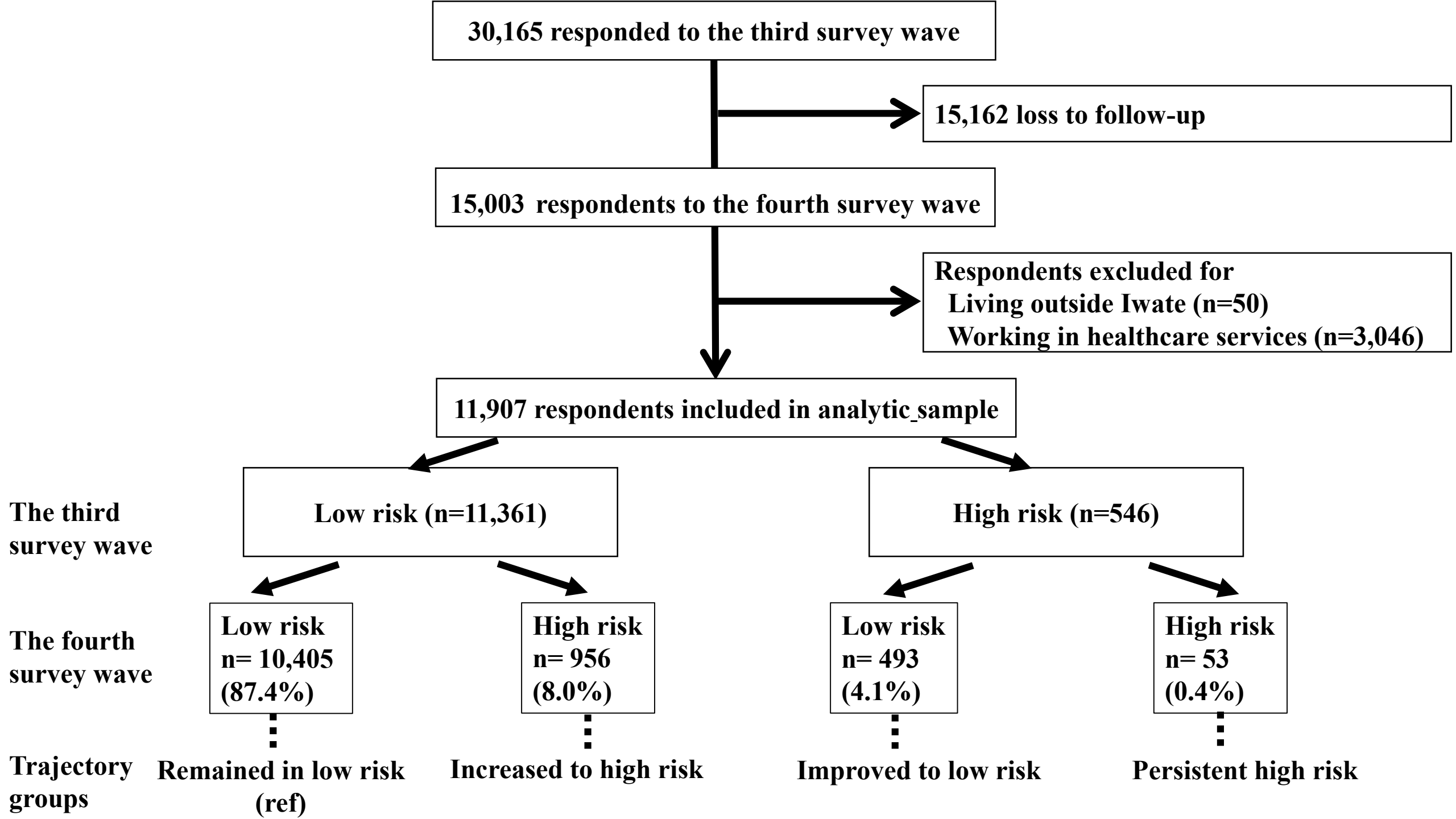

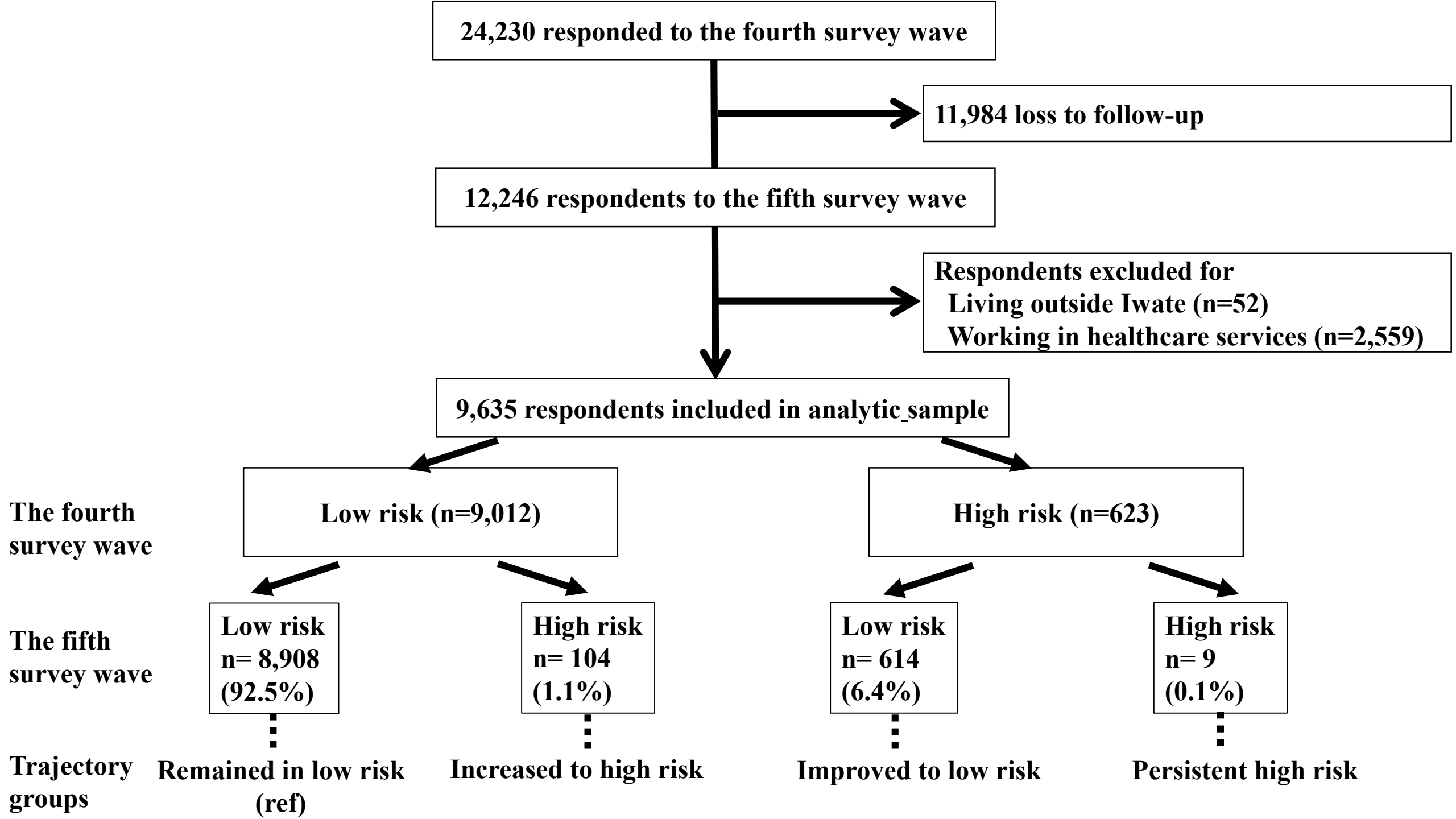

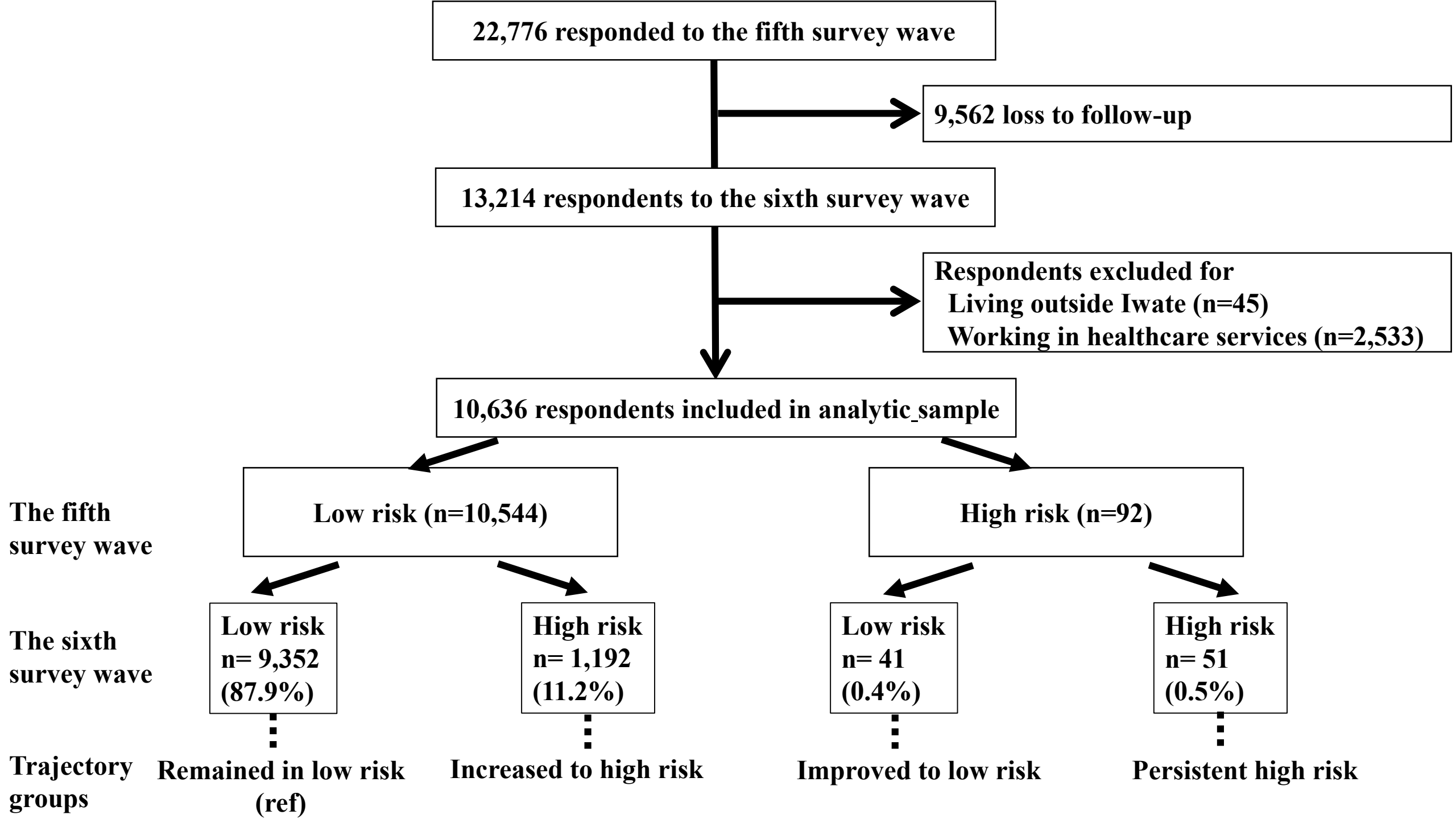

Supplement: Supplementary file 3 — Supplementary Fig. 1. Flow chart of the selection of respondents. Out of 170000 registered residents, Out of 170,000 registered residents, 9,385, 11,907, 9, 635, and 10,636 individuals were selected in the four analytic samples, respectively.Multimedia component 3 [file mmc3.pdf]
